# Molecular and functional aspects of menstruation in the macaque

**DOI:** 10.1007/s11154-012-9225-5

**Published:** 2012-10-30

**Authors:** Robert M. Brenner, Ov D. Slayden

**Affiliations:** Division of Reproductive & Developmental Sciences, Oregon National Primate Research Center, Oregon Health and Science University, 505 NW 185th Ave, Beaverton, OR USA

**Keywords:** Macaque, Menstruation, Model, Hormonal control, Menstrual blood loss, Endometrium, Cervix, Heavy menstrual bleeding

## Abstract

Much of our understanding of the molecular control of menstruation arises from laboratory models that experimentally recapitulate some, but not all, aspects of uterine bleeding observed in women. These models include: *in vitro* culture of endometrial explants or isolated endometrial cells, transplantation of human endometrial tissue into immunodeficient mice and the induction of endometrial breakdown in appropriately pretreated mice. Each of these models has contributed to our understanding of molecular and cellular mechanisms of menstruation, but nonhuman primates, especially macaques, are the animal model of choice for evaluating therapies for menstrual disorders. In this chapter we review some basic aspects of menstruation, with special emphasis on the macaque model and its relevance to the clinical issues of irregular and heavy menstrual bleeding (HMB).

## Introduction

Much of our understanding of the molecular control of menstruation arises from laboratory models that experimentally recapitulate some, but not all, aspects of uterine bleeding observed in women. These models include: *in vitro* culture of endometrial explants or isolated endometrial cells [1–3], transplantation of human endometrial tissue into immunodeficient mice [4], and the induction of endometrial breakdown in appropriately pretreated mice [5–8]. Each of these models has contributed to our understanding of molecular and cellular mechanisms of menstruation, but nonhuman primates, especially macaques, are the animal model of choice for evaluating therapies for menstrual disorders. In this chapter we review some basic aspects of menstruation, with special emphasis on the macaque model and its relevance to the clinical issues of heavy and irregular menstrual bleeding.

Menstrual disorders including heavy menstrual bleeding (HMB), irregular uterine bleeding, and painful menstruation are common problems experienced by many women. HMB is considered as excessive menstrual blood loss, prolonged more than 8 days, which interferes with the woman’s physical, emotional, social and material quality of life, and which can occur alone or in combination with other symptoms [9]. Irregular menstruation is characterized by cycles that vary more than 8 days in length. HMB affects approximately one-third of reproductive aged women and although heavy bleeding can be associated with endometriosis and uterine fibroids, greater than 80 % of patients with HMB exhibit no clear underlying cause [10]. Idiopathic HMB is one of the most common gynecologic conditions requiring hospitalization, and 20–30 % of all hysterectomies are performed to stop abnormal bleeding. Expansion of medical therapies for HMB would greatly benefit the health of many women who cannot or do not wish to undergo surgery [11].

## Phylogenetic considerations

**Fig. 1:**
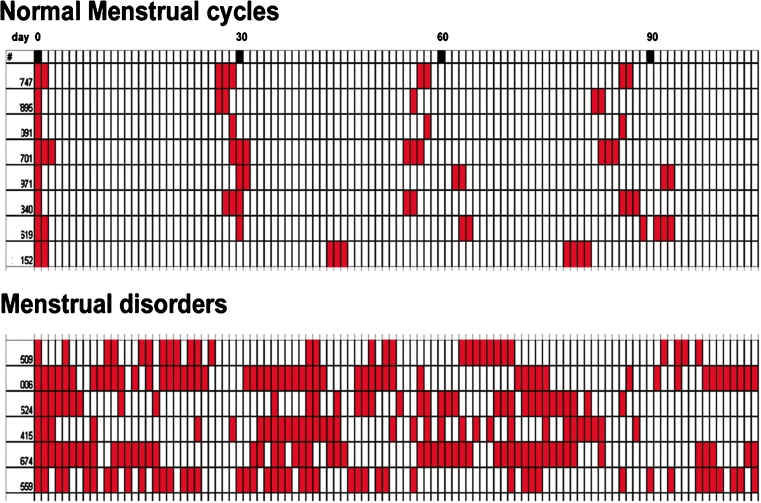
Comparison of normal versus disordered and heavy menstrual bleeding (HMB) in rhesus macaques. *Top panel*: bleeding patterns of 8 animals with normal cycles. *Bottom panel*: bleeding patterns of 6 animals with abnormal cycles. Each row shows the days of bleeding of one animal followed for 110 days, aligned by the first day of bleeding. The *lower panel* shows the erratic bleeding patterns of monkeys with menstrual disorders including irregular bleeding and HMB

Periodic uterine bleeding is reported for a small number of non-primate mammals, including some species of tree shrew [12]. However, cyclic, hormonally regulated shedding of the endometrium, characteristic of true menstruation, is found only in a few species of bats [13, 14] and in primates, including Homo sapiens, the Great Apes (Hominidae), Lesser Apes (Hylobatidae) and Old World monkeys (Cercopithecidae) [15]. In these primate species, menstruation follows the fall in progesterone levels as the corpus luteum regresses at the end of the ovarian cycle, and cycle lengths are counted from the first day of menstrual bleeding. Menstrual cycle length varies slightly between primate species, from about 28 days in women and rhesus macaques to about 37 days in chimpanzees. Rhesus (*Macaca mulatta*), cynomolgus *(Macaca fascicularis)*, and pigtailed macaques (*Macaca nemestrina)* display approximately 28 day menstrual cycles, similar to those of women [16–18]. Rhesus macaques are the most widely used nonhuman primate model for menstrual bleeding studies [19–21]. Macaques can be trained to allow daily vaginal swabs to detect menstrual bleeding [21] and can be fitted with tampons [22] for more quantitative analysis of menstrual blood loss. Naturally cycling macaques may display spontaneous menstrual disorders, including irregular bleeding and HMB (Fig. 1), making them ideal for clinically relevant studies. Further, macaques can be ovariectomized and treated with ovarian steroids to induce artificial menstrual cycles. Induced cycles reduce the variability inherent in natural cycles, and permit endometrial sampling during precisely defined premenstrual, menstrual and postmenstrual phases.

## Anatomical considerations

**Fig. 2:**
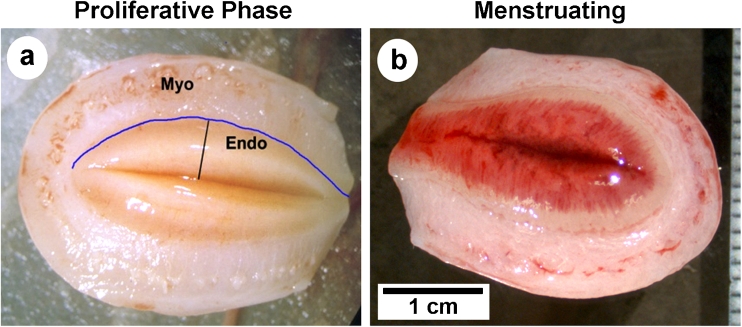
Comparison of rhesus uterus in proliferative versus menstrual phase. Freshly removed uteri were cut in half along the fundal-cervical axis and photographed with macro lenses. **a** a *dark line marks* the endometrial-myometrial border and another line delineates the plane along which sections of the endometrium were cut. This specimen was taken on day 14 of the induced proliferative phase. Endo = endometrium. Myo = myometrium. **b** on day 2 of the menstrual cycle, bleeding is restricted to the upper third of the endometrium. The basalis and the lowest part of the functionalis does not bleed or slough. Scale bar = 1 cm; applies to both images

**Fig. 3:**
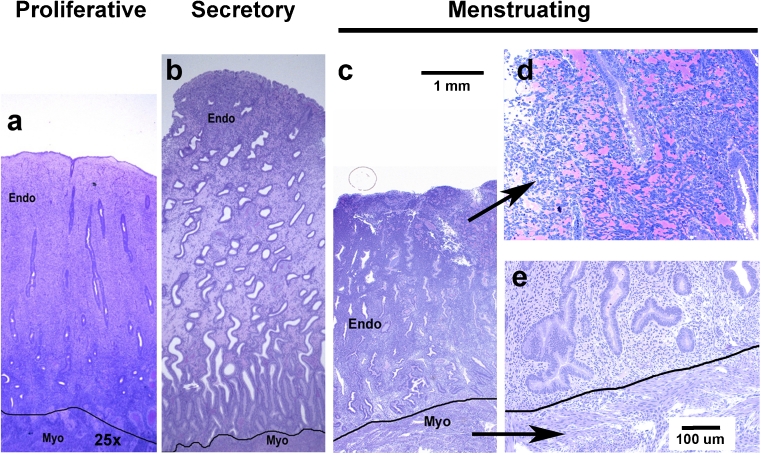
Histology of the rhesus endometrium in the proliferative, secretory and menstrual phases. Histological sections of the endometrium were cut on a plane running from the luminal to the myometrial border. Straight versus tortuous glands are evident in (**a** versus **b**). Sections taken during the menstrual phase (**c**–**e**) show that menstrual breakdown is only evident in the functionalis (**c**–**d**) not the basalis (**e**). Scale bar for a–c = 1 mm; scale bar for d–e = 100 μm

The macaque uterus is morphologically similar to the human uterus, consisting of the fundus (dome-shaped top), corpus (body), and isthmus (neck) leading to the cervix. The endometrium lines the uterine cavity and is surrounded by the muscular wall or myometrium (Fig. 2a). In women [23] and macaques [24] the endometrium has 4 layers or “zones” extending from the luminal surface to the myometrial border. In that classification, the luminal epithelium and an underlying band of stromal cells is defined as Zone I. Slightly deeper, Zone II contains glands that run perpendicular to the surface. Zones I and II are sometimes referred to as the “compacta” in women and consist of densely packed stromal cells around the straight necks of the glands. Deeper still, Zone III, sometimes referred to as the spongiosa in women, contains glands that are branched. The deepest zone, Zone IV, the basalis, is adjacent to the myometrium, where the glands terminate (Fig. 3). The upper zones (e.g. the compacta and upper spongiosa) are also referred to as the functionalis, or functional layer, as opposed to the basalis, or basal layer. The functionalis, which undergoes secretory transformation under the influence of progesterone, derives its blood supply from specialized spiral arteries rather than the basal arteries that supply the deepest, basal zone [25]. The spiral arteries are unique endometrial vessels in menstruating primates that undergo hormonally driven regeneration after menstruation in each cycle. In the secretory phase, progesterone stimulates spiral artery hypertrophy, and during a fertile cycle these arteries play an important role in embryo implantation. Abnormal spiral arteriogenesis can contribute to pregnancy failure and may lead to breakthrough bleeding and HMB [26].

**Fig. 4:**
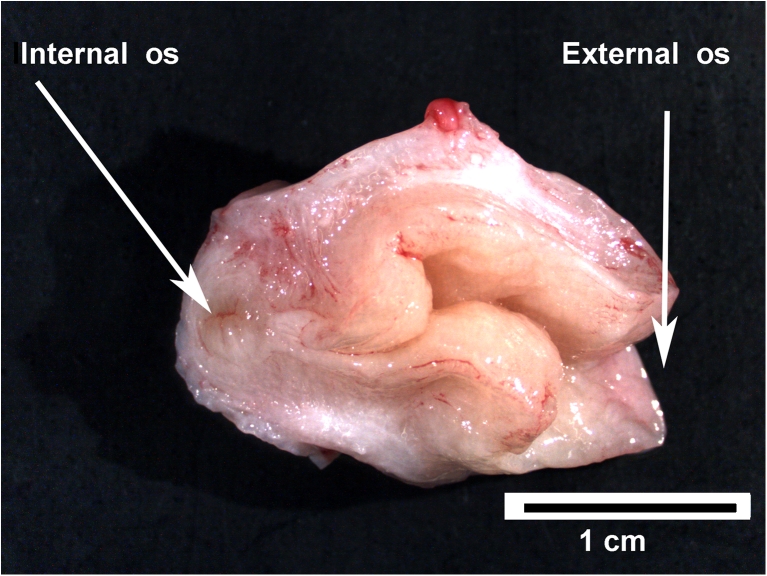
The rhesus macaque cervix. A cervix was freshly removed from a rhesus macaque at midcycle and cut longitudinally along the axis from the External os to Internal os. A projection of the cervical wall (colliculum) forces the cervical canal into a Z-shaped path. Scale bar = 1 cm

While the macaque uterus is strikingly similar to that of most other Old World nonhuman primates, the anatomy of the cervix varies greatly between primate species [27]. In baboons [28] ( as in women) the cervical canal is relatively free from obstruction, which facilitates hysteroscopy for the assessment of endometrial bleeding and collection of samples by curettage [29–31]. However, in macaques there is a prominent colliculum (Fig. 4) that obstructs passage of instruments into the uterine cavity [32]. Estrogen stimulation induces increased curvature that further restricts access to the cervical canal.

## The macaque menstrual cycle

Central regulation of female sexual maturation, follicular development, ovulation, and ovarian hormone secretion in primates involves interplay between the hypothalamus, pituitary and ovary, relationships that have been reviewed extensively [33–35]. In primates, ovarian cyclicity is not dependent on endocrine feedback from the uterus. Estradiol (E) and progesterone are the only ovarian factors required for induction of menstruation [36–38]. Briefly, the menstrual cycle can be divided into three phases: the menstrual phase, the follicular (or proliferative) phase, and the luteal (or secretory) phase. Estradiol secreted by the ovary during the follicular phase stimulates cell proliferation in both the endometrial glands and stroma [39]. In macaques the length of the proliferative phase of the natural cycle is normally 10–14 days. After ovulation, progesterone levels rise during the luteal phase [39] of the cycle, which usually lasts for 12 to 14 days. If pregnancy does not occur, the corpus luteum regresses and the decline of progesterone triggers menstruation .

In various studies, we induced cycles in ovariectomized macaques by treating the animals with Silastic capsules that release E and progesterone [40]. Briefly, animals are treated first with E alone, which stimulates an artificial proliferative phase, characterized by tubular endometrial glands and abundant proliferating cells in the glandular epithelium. After 14 days of E priming, addition of a progesterone capsule induces an artificial secretory phase, which becomes evident by day 3 of progesterone treatment. Progesterone acts to gradually suppress cell proliferation in the glandular epithelium, which thickens, and the glands become saw-toothed and sacculated in appearance, with shrunken nuclei and jagged apical surfaces. In the macaque, the early secretory phase is marked by a striking up regulation of cell proliferation in the glands of the basalis zone [41, 42]. Withdrawal of the progesterone capsule (with E left in place) completes the cycle and induces menses on days 2–5 of the next cycle. We refer to this 2–5 days interval as the luteal-follicular transition (LFT) [43, 44].

The effects of ovarian steroid hormones on estrogen and progesterone receptors have been extensively reviewed [45–47]. Briefly, during the proliferative phase, E stimulates expression of estrogen receptor-1 (ESR-1) and progesterone receptor (PGR) in the endometrial glandular epithelium and stromal fibroblasts. During the secretory phase, progesterone suppresses ESR-1 in glands and stroma of the functionalis zone, while suppressing PGR only in the glands, not the stroma. Thus, progesterone can act through PR in the stromal fibroblasts to control expression of tissue degrading enzymes, growth factors, and extracellular matrix molecules. The basalis zone is unique in retaining both ESR-1 and PGR in both the glands and stroma during the secretory phase. The vascular endothelium in all zones fails to expresses ESR-1 or PGR but does express ESR-2 (ER-beta [45, 48]).

Endometrial angiogenesis, vascular growth, and vasoconstriction [49, 50] have been extensively studied in macaques [51, 52] because the vessels of the upper functionalis are shed and regenerated after menstruation. Endothelial cell proliferation peaks in small vessels during the proliferative phase [51, 53]. After ovulation, the endometrial spiral arteries hypertrophy [42], and provide increased blood flow [54] to the upper zones where embryo implantation and early placentation occur [55]. The effects of progesterone on the spiral arteries are probably mediated by factors arising from PGR-positive stromal cells or through novel non-nuclear receptors [56]. At the end of a nonfertile cycle the fall in progesterone triggers waves of vasoconstriction [57] of the spiral arteries typically followed 4 to 24 h later by menstruation.

## Histological aspects of menstrual breakdown

On day 28 of an induced cycle, the endometrium appears thickened with stromal cell hypertrophy, tissue edema and tortuous glands in the functionalis zone. Twenty four hours after progesterone withdrawal the endometrium shrinks dramatically and the stroma becomes highly compacted. By day 2 the uppermost third of the endometrium undergoes extensive fragmentation accompanied by hemorrhage and sloughing (Fig. 2b). The deeper functionalis and basalis zones show no signs of fragmentation, but the glandular epithelial cells in the basalis undergo extensive apoptotic cell death during days 2–6.

Overt menstrual bleeding typically ends on day 4–6, and around day 5 mitotic activity begins in the necks of the surviving glands [44]. By day 8 there is increased endometrial growth of the glands as well as maximal endothelial cell proliferation [51]. Structural regrowth of the endometrium in the follicular phase appears complete by day 10–14, at which time the endometrium consists of straight, nonsacculated tubular glands encompassed within a moderately loose stroma.

## Effects of progesterone withdrawal on endometrial matrix metalloproteinases

The decline of progesterone at the end of the cycle is followed by increased expression of tissue degrading matrix metalloproteinase (MMP) enzymes [19, 58]. The MMPs have the enzymatic capability to mediate tissue dissolution in the upper functionalis zones and are considered primary effectors of menstrual sloughing [59, 60]. MMPs are a multigene family of enzymes that require zinc for their activation [61] . Secreted as proenzymes, the MMPs undergo activation to a catalytically active form in the endometrium. Enzyme activation is regulated by specific endogenous tissue inhibitors of metalloproteinases (TIMPs) [61] that are also expressed in the endometrium [62, 63]. *In vitro* studies on endometrial stromal cells in culture demonstrate that progesterone suppresses expression of several MMPs [59, 64]. Paradoxically, progesterone receptor consensus sequences have not been identified on the MMPs associated with menstruation. Moreover, in the macaque, endometrial expression of several MMPs declines rapidly after menstruation, well before progesterone levels increase [19]. Therefore, progesterone mediated regulation of MMPs appears to be either indirect through paracrine/autocrine factors, or through non-traditional mechanisms. Because of the focal nature of menstrual breakdown and the restriction of MMP expression to the mid and upper functionalis zones, it has been suggested that local factors such as specific cytokines can regulate endometrial MMPs. For instance, TGF-beta has been identified as a mediator of MMP-7 suppression by progesterone [65]. Also, endometrial bleeding associated factor (ebaf; lefty-A) has been identified as an inhibitor of TGF-beta [66], and lefty-A can induce expression of several MMPs in human endometrial stromal cells. Because progesterone can still suppress MMPs upregulated by lefty-A *in vitro* [67], the mechanism through which progesterone regulates endometrial MMPs remains unclear.

**Fig. 5:**
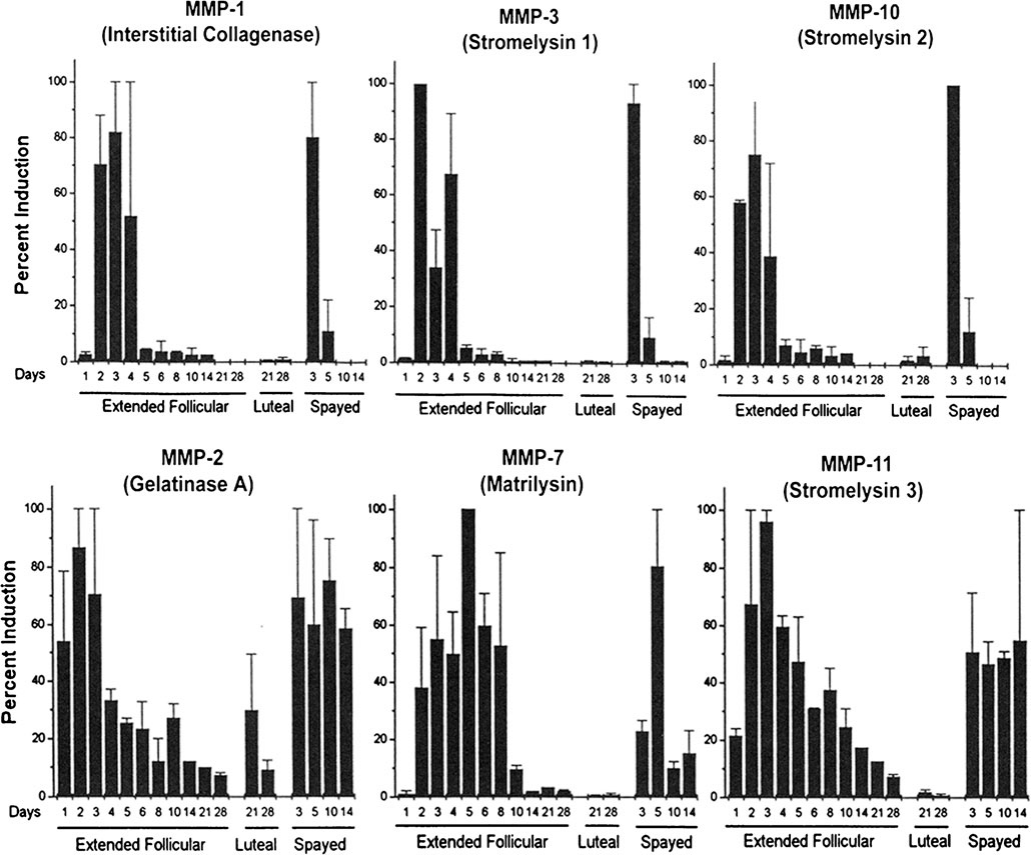
mRNA expression of various MMPs in the rhesus menstrual cycle. Plots of northern hybridization data reveal two patterns of MMP mRNA expression. *Top Row*: Certain MMPs were strongly expressed during the menstrual phase and declined to minimal levels by days 5–6. *Bottom Row*: Other MMPs peaked similarly during menstruation but declined much more slowly

**Fig. 6:**
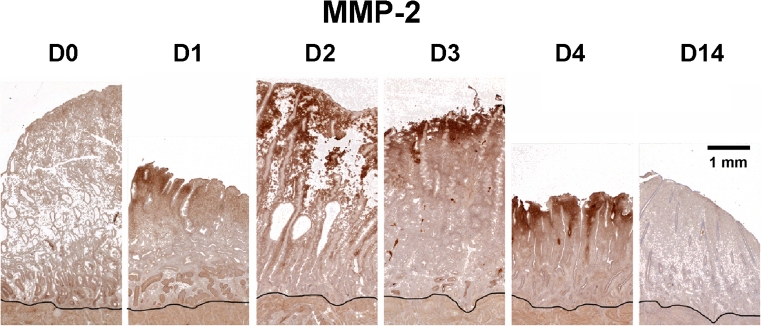
Immunohistochemistry of MMP-2 during the proliferative phase. Endometrial sections were stained for MMP-2 on days (D) 0,1,2,3,4 and 14 after P withdrawal. The *black line* marks the myometrial border. *Strong dark brown* staining for MMP-2 protein was confined to the upper functionalis zone during the menstrual phase (D1-4). The staining became nondetectable by day 14. Scale bar = 1 mm; applies to all images

In earlier studies we documented the timing of menstrual sloughing and the expression of various MMP enzymes in rhesus and pigtailed macaques [19, 40]. For these studies the animals were treated with E and progesterone to induce artificial menstrual cycles, and the uterus was removed from the animals on days 1,2,3,4,5,6,8,10,14,21 and 28 of the cycle. In this design, day 0 represents the day of progesterone implant removal. In other animals we removed both the E and the progesterone releasing capsules to determine whether the absence of E would affect MMP expression. These latter animals were referred to as either spayed or hormone-deprived. In each case, samples of endometrium were prepared for immunocytochemistry and analysis of MMP mRNA expression. In general, the expression of endometrial MMPs *in vivo* during the menstrual cycle of the rhesus macaque was very similar to that reported for women. Most of the MMPs were expressed at relatively high levels just before and during menstrual breakdown (Fig. 5). Immunolocalization studies revealed strong staining for MMP-1, MMP-2, and MMP-3, localized primarily to the stromal cells of the fragmenting zones, with intense staining in the cytoplasm of stromal cells at the basement membrane of regressing spiral arteries. Immnocytochemical staining patterns of MMP-2 are illustrated in Fig. 6. MMP-7 was initially expressed by the glands immediately below the fragmenting zone, and later in the upper glands as the endometrium entered the repair phase. MMP-7 localization became stronger as MMP-2 and MMP-3 staining waned in the early follicular phase. Tissue inhibitor of metalloproteinases-1 (TIMP-1) also increased, at both the protein and gene expression level, by cycle day 2. This increase was especially apparent in the smooth muscle and perivascular stroma of the spiral arteries and in large numbers of stromal cells in the fragmenting and sloughing endometrial regions. All these effects were similar in the presence and absence of E.

Several studies have proposed that induction of menstruation occurs in two phases [21, 68, 69]. In the first phase, progesterone withdrawal initiates up regulation of specific paracrine regulators, especially those secreted by the perivascular cells of the spiral arteries. Once these regulators are fully expressed, a second phase dominated by tissue degrading enzymes develops. A corollary of this hypothesis is that the first, but not the second phase can be interrupted by re-elevation of progesterone [21]. This suggests that progesterone withdrawal is followed by a “critical period” after which tissue breakdown, sloughing and bleeding become inevitable. To further define the critical period in macaques, we induced cycles as described above and, at the end of the luteal phase, withdrew progesterone at time 0 and then either replaced progesterone (or not) 12, 24, or 48 h later. Uteri from experimental (progesterone-replaced) and control (not replaced) animals were collected 36, 48 and 72 h after progesterone withdrawal (*n* = 3/interval). Strong immunocytochemical staining for MMP-1 and MMP-2 was evident in controls at 36, 48 and 72 h. Progesterone replacement at 12–24 h after P withdrawal (the critical period), blocked menses and blocked expression of both MMPs. This confirms a role for both MMP-1 and MMP-2 in the initiation of menstruation. However, progesterone replacement after the critical period failed to suppress menses and failed to suppress MMP2, though MMP-1 was suppressed. Therefore, some MMPs may be more important for initiating menses, and others for sustaining menstrual breakdown. Our results confirm and extend the view that there is a physiologically significant critical period underlying the menstrual process. The particular role that each MMP plays in the menstrual cascade remains to be determined.

## Vascular events associated with menstruation

Much of our understanding of menstruation arises from classical studies by Markee [49]. Markee transplanted rhesus macaque endometrium to the anterior chamber of the eye and visualized the events of menstruation directly. He observed pulses of vasoconstriction in the spiral arteries after progesterone withdrawal that could lead to localized ischemic hypoxia in the superficial zones. Vascular endothelial growth factor (VEGF) is reported to be upregulated by hypoxia in the human endometrium [70], and in our macaque studies, stromal and glandular VEGF were dramatically up regulated premenstrually. VEGF may interact with several receptors, including VEGFR-1 and VEGFR-2 which are located on the membranes of endothelial cells [71, 72]. However, VEGFR-2 expression was also upregulated by progesterone withdrawal in the stromal cells of the functionalis zone. Whether VEGF interacts with VEGFR-2 in these stromal cells after progesterone withdrawal remains to be determined.

**Fig. 7:**
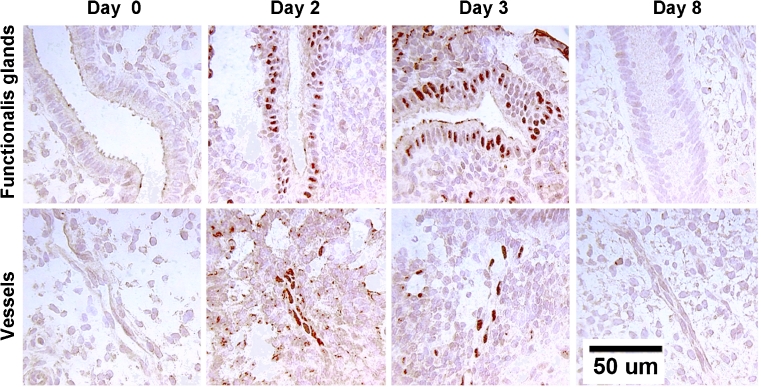
Immunohistochemistry of hypoxia inducible factor in the macaque endometrium. Endometrial sections were stained for hypoxia inducible factor protein on days 0, 2,3, and 8 after P withdrawal. A mouse monoclonal antibody against HIF-1α at a concentration of 1:1000 (Novus Biologicals) was used. Nuclear HIF-1α staining was low on day 0, increased strikingly on days 1–3 of the cycle and then declined to undetectable by day 8. These increases occurred primarily in the glands and the small blood vessels of the upper functionalis zone, presumably in response to local hypoxia. Scale bar = 50 um; applies to all images

We also examined the expression and localization of hypoxia inducible factor 1 (HIF-1α) in the rhesus macaque endometrium during the menstrual-early proliferative stage of an induced cycle. HIF-1α is a nuclear protein that mediates the effects of hypoxia on gene expression. Artificial cycles were induced in 26 ovariectomized rhesus macaques. After 14 days the P implant was removed and the uterus was collected from 2 animals each on days 0, 1, 2, 3, 4, 5, 6, 8 and 14 (P withdrawn). In another group, the P and E implants were both withdrawn on day 0 and the uteri were collected on days 1, 2, 3, and 14 (hormone deprived). Menses occurred in both groups on days 2–5. Endometrial samples were analyzed for HIF-1 mRNA by Northern blot and *in situ* hybridization . Northern blots revealed that HIF-1α mRNA levels were low on day 0, increased strikingly on days 1–3 of the cycle and then declined. Similarly, in the hormone-deprived group, the HIF-1α mRNA increased on days 1 and 2 and then declined. These increases occurred primarily in the glands and the small blood vessels of the upper functionalis zone. Grain counts of the ISH preparations showed a 6.6 fold increase in the blood vessels and a 1 .4 fold increase in the glands over baseline during days 1 and 2 followed by a decline. Immunocytochemical staining of the HIF-1α nuclear protein showed a similar pattern of expression (Fig. 7). In sum, HIF-1α was strikingly elevated in endometrial vessels and glands in the functionalis on days 1–3 after P withdrawal whether or not E was present. These data support a role for hypoxia, mediated by HIF-1α, in the early phases of the menstrual cascade.

## Endometrial repair

Much more is known about the destructive sloughing of the menstrual process than the reconstructive processes involved in menstrual repair. These include re-epithelialization, surface healing and cessation of bleeding. Immediately after menstruation the endometrial surface is ragged and torn with multiple gland openings surrounded by denuded stromal elements [73]. Healing of this denuded stromal surface begins with the transformation of the uppermost gland cells into a migratory phenotype. Cells move from the necks of the glands, spread out over the raw suface, meet migrating cells from other glands and combine with them to form a new luminal surface. By day 5, estrogen driven mitotic activity begins in the necks of the glands [44].

The underlying cellular and molecular mechanisms that support this form of endometrial “wound healing” are not understood. In other wounded tissues, particularly skin [74], a variety of molecules including fibronectin, various collagens, laminin and vitronectin are involved in the healing process . Fibronectin is a large fibrillar glycoprotein, secreted usually as a homodimer, composed of two ~250 kDa monomers linked together by a pair of disulphide bonds. Interactions between fibronectin and specific integrins are known to enhance cell adhesion and migration during wound healing [75–77]. In a recent study, [78], we analyzed samples of rhesus macaque endometrium with pathway-focused arrays of human genes associated with the extracellular matrix and found that fibronectin and integrin β1 transcripts were dramatically increased during menstruation and repair, compared to the late secretory phase of the cycle. The results showed that fibronectin and specific fibronectin receptors increased dramatically during menstruation and repair and that these molecules were only expressed in the uppermost endometrial zones. Additional research on the related factors in the extracellular matrix is needed to further define endometrial “wound healing”, a process that is highly relevant to the clinical problem of uncontrolled endometrial bleeding.

## Studies of menstrual blood loss

**Fig. 8:**
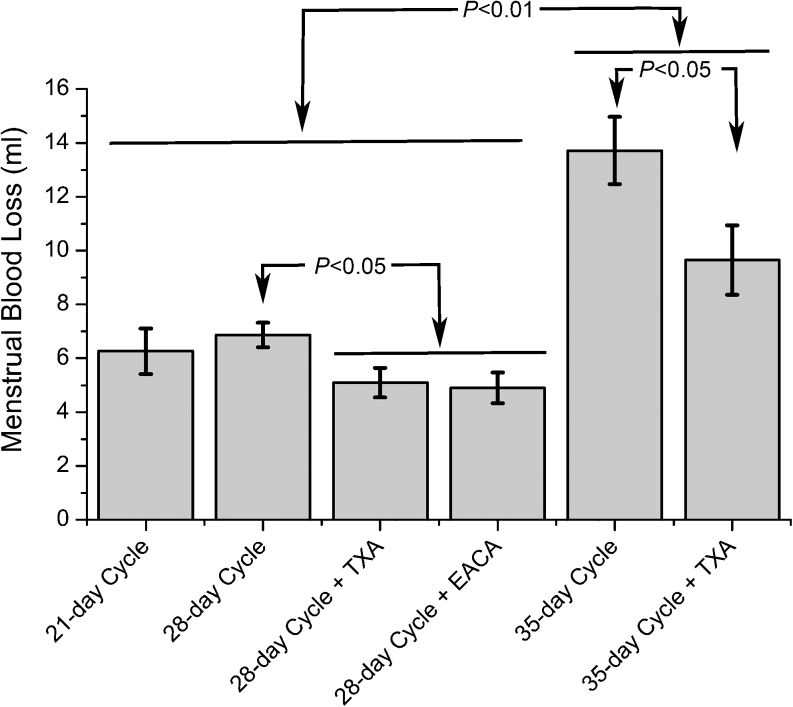
Mean menstrual blood loss over 6 days in artificially cycled macaques. Menstruation peaked in all animals on day 3 after progesterone withdrawal. Short (21 days) menstrual cycles had no significant effect on total menstrual blood loss compared to normal length (28 days) cycles, but treatment with artificially amplified cycles (35 days) in which the secretory phase had elevated and prolonged exposure to progesterone resulted in increased bleeding (*P* < 0.01). Treatment with antifibrinolytics including tranexamic acid (TXA; 75 mg/kg/day) or ε-aminocaproic acid (EACA; 100 mg/kg/day) significantly reduced menstrual blood loss (*P* < 0.05). Values were compared statistically by Analysis of Variance and means were compared by Fisher’s protected LSD

Recently we utilized a rigorous method for measuring menstrual blood loss in rhesus macaques. Macaque-size tampons were devised and used to collect the menstrual flow. Tampons were removed daily, vacuum dried and mixed with 5 % sodium hydroxide. The resulting alkaline haematin extract was quantified spectrophotometrically at 564 nm on Elisa plates. Average total blood loss during the menstrual cycle in 11 animals was 5.87 +/− 0.6 ml (over 6 days). Removal of the tampons daily revealed that menstrual blood loss peaked in all the animals on day 3 after progesterone withdrawal. To assess the effects of ovarian hormones on menstrual blood loss, we manipulated both the serum level of progesterone and the length of the secretory phase. Short menstrual cycles were created by treating the animals with progesterone (5–6 ng/ml) for 7 days. Normal menstrual cycles had similar levels of progesterone for 14 days. Extended 35 day menstrual cycles had 2 progesterone implants that produced 11–13 ng/ml for 21 days. Interestingly, treatment with short cycles had no significant effect on total menstrual blood loss compared to normal length cycles (Fig. 8). In contrast, treatment with extended cycles with elevated progesterone resulted in significantly greater menstrual blood loss (Fig. 8). We also treated animals with two anti-fibrinolytic therapies, tranexamic acid (TXA) or ε-aminocaproic acid (EACA;) beginning on cycle day 0 for 5 days. Both significantly reduced menstrual blood loss. We will evaluate alternative therapies for ameliorating menstrual blood loss in rhesus macaques with a similar quantitative approach.

## Conclusion

In sum, the rhesus macaque has contributed significantly to our understanding of both the control and the nature of menstruation. Many novel target molecules associated with menstruation, such as the MMPs, VEGF, TIMPs and components of the extracellular matrix, have been identified. Exploration of these and other target molecules should lead to a deeper understanding of severe menstrual blood loss, and may lead to new suppressive therapies for HMB. Our laboratory remains committed to this goal.

## References

[1] Lockwood CJ, Krikun G, Hausknecht VA, Papp C, Schatz F (1998). Matrix metalloproteinase and matrix metalloproteinase inhibitor expression in endometrial stromal cells during progestin-initiated decidualization and menstruation-related progestin withdrawal. Endocrinology.

[2] Marbaix E, Vekemans M, Galant C, Rigot V, Lemoine P, Dubois D (2000). Circulating sex hormones and endometrial stromelysin-1 (matrix metalloproteinase-3) at the start of bleeding episodes in levonorgestrel-implant users. Hum Reprod.

[3] Salamonsen LA, Woolley DE (1996). Matrix metalloproteinases and their tissue inhibitors in endometrial remodelling and menstruation. Reprod Med Rev.

[4] Guo Y, He B, Xu X, Wang J (2011). Comprehensive analysis of leukocytes, vascularization and matrix metalloproteinases in human menstrual xenograft model. PLoS One.

[5] Brasted M, White CA, Kennedy TG, Salamonsen LA (2003). Mimicking the events of menstruation in the murine uterus. Biol Reprod.

[6] Kaitu’u-Lino TJ, Phillips DJ, Morison NB, Salamonsen LA (2009). A new role for activin in endometrial repair after menses. Endocrinology.

[7] Kaitu’u TJ, Shen J, Zhang J, Morison NB, Salamonsen LA (2005). Matrix metalloproteinases in endometrial breakdown and repair: functional significance in a mouse model. Biol Reprod.

[8] Rudolph M, Rudolph M, Docke WD, Muller A, Menning A, Rose L, Zollner TM (2012). Induction of overt menstruation in intact mice. PLoS One.

[9] Fraser IS, Critchley HO, Broder M, Munro MG (2011). The FIGO recommendations on terminologies and definitions for normal and abnormal uterine bleeding. Semin Reprod Med.

[10] Naoulou B, Tsai MC (2012). Efficacy of tranexamic acid in the treatment of idiopathic and non-functional heavy menstrual bleeding: a systematic review. Acta Obstet Gynecol Scand.

[11] Warner P, Critchley HO, Lumsden MA, Campbell-Brown M, Douglas A, Murray G (2001). Referral for menstrual problems: cross sectional survey of symptoms, reasons for referral, and management. BMJ.

[12] Finn CA (1987). Why do women and some other primates menstruate?. Perspect Biol Med.

[13] Rasweiler JJ, de Bonilla H (1992). Menstruation in short-tailed fruit bats (Carollia spp.). J Reprod Fertil.

[14] Rasweiler JJ (1991). Spontaneous decidual reactions and menstruation in the black mastiff bat, Molossus ater. Am J Anat.

[15] Martin RD (2007). The evolution of human reproduction: a primatological perspective. Am J Phys Anthropol Suppl.

[16] Brenner RM, West NB, McClellan MC (1990). Estrogen and progestin receptors in the reproductive tract of male and female primates. Biol Reprod.

[17] Chandrashekar V, Wolf RC, Dierschke DJ, Sholl SA, Bridson WE, Clark JR (1980). Serum progesterone and corpus luteum function in pregnant pigtailed monkeys (Macaca nemestrina). Steroids.

[18] Eaton GG, Resko JA (1974). Ovarian hormones and sexual behavior in *Macaca nemestrina*. J Comp Physiol Psychol.

[19] Rudolph-Owen LA, Slayden OD, Matrisian LM, Brenner RM (1998). Matrix metalloproteinase expression in *Macaca mulatta* endometrium: evidence for zone-specific regulatory tissue gradients. Biol Reprod.

[20] Slayden OD, Chwalisz K, Brenner RM (2001). Reversible suppression of menstruation with progesterone antagonists in rhesus macaques. Hum Reprod.

[21] Slayden OD, Brenner RM. A critical period of progesterone withdrawal precedes menstruation in macaques. Reprod Biol Endocrinol. 2006;S6 Suppl 1. 10.1186/1477-7827-4-S1-S6 PMC1775066 17118170

[22] Shaw ST, Elsahwi SY, Moyer DL (1972). Menstrual blood quantitation in the rhesus monkey: an experimental tool for improving intrauterine contraceptive devices (IUDS). Fertil Steril.

[23] Bartelmez GW (1957). The phases of the menstrual cycle and their interpretation in terms of the pregnancy cycle. Am J Obstet Gynecol.

[24] Bartelmez GW (1951). Cyclic changes in the endometrium of the rhesus monkey (*Macaca mulatta*). Contrib Embryol.

[25] Bartelmez GW (1957). The form and the functions of the uterine blood vessels in the rhesus monkey. Contrib Embryol.

[26] Rogers PA, Abberton KM (2003). Endometrial arteriogenesis: vascular smooth muscle cell proliferation and differentiation during the menstrual cycle and changes associated with endometrial bleeding disorders. Microsc Res Tech.

[27] Demers LM, Macdonald GJ, Hertig AT, King NW, MacKey JJ (1972). The cervix uteri in *Macaca mulatta, Macaca arctoides,* and *Macaca fascicularis*—A comparative anatomic study with special reference to *Macaca arctoides* as a unique model for endometrial study. Fertil Steril.

[28] Nyachieo A, Chai DC, Deprest J, Mwenda JM, D’Hooghe TM (2007). The baboon as a research model for the study of endometrial biology, uterine receptivity and embryo implantation. Gynecol Obstet Investig.

[29] Agdi M, Tulandi T (2010). Minimally invasive approach for myomectomy. Semin Reprod Med.

[30] Hickey M, Dwarte D, Fraser IS (2000). Superficial endometrial vascular fragility in Norplant users and in women with ovulatory dysfunctional uterine bleeding. Hum Reprod.

[31] Lethaby A, Hickey M, Garry R. Endometrial destruction techniques for heavy menstrual bleeding. Cochrane Database Syst Rev. 2005;14:CD001501. 10.1002/14651858.CD001501.pub2 16235284

[32] Davis RH, Schneider HP (1975). Cast of the Z-shaped cervical canal of the uterus of the rhesus monkey. Lab Anim Sci.

[33] Weinbauer GF, Niehoff M, Niehaus M, Srivastav S, Fuchs A, van Esch E (2008). Physiology and endocrinology of the ovarian cycle in macaques. Toxicol Pathol.

[34] Pau K-YF, Berria M, Hess DL, Spies HG (1993). Preovulatory gonadotropin-releasing hormone surge in ovarian-intact rhesus macaques. Endocrinology.

[35] Chaffin CL, Schwinof KM, Stouffer RL (2001). Gonadotropin and steroid control of granulosa cell proliferation during the periovulatory interval in rhesus monkeys. Biol Reprod.

[36] Hisaw FL, Hisaw FL, Young WC (1961). Action of estrogen and progesterone on the reproductive tract of lower primates. Sex and Internal Secretions.

[37] Okulicz WC, Ace CI (2003). Temporal regulation of gene expression during the expected window of receptivity in the rhesus monkey endometrium. Biol Reprod.

[38] Hodgen GD (1983). Surrogate embryo transfer combined with estrogen-progesterone therapy in monkeys. JAMA.

[39] Brenner RM, Slayden OD, Knobil E, Neill JD (1994). Cyclic changes in the primate oviduct and endometrium. The Physiology of Reproduction.

[40] Brenner RM, Rudolph L, Matrisian L, Slayden OD (1996). Non-human primate models: artificial menstrual cycles, endometrial matrix metalloproteinases and s.c. endometrial grafts. Hum Reprod.

[41] Okulicz WC, Balsamo M, Tast J (1993). Progesterone regulation of endometrial estrogen receptor and cell proliferation during the late proliferative and secretory phase in artificial menstrual cycles in the rhesus monkey. Biol Reprod.

[42] Slayden OD, Keator CS (2007). Role of progesterone in nonhuman primate implantation. Semin Reprod Med.

[43] McClellan M, West NB, Brenner RM (1986). Immunocytochemical localization of estrogen receptors in the macaque endometrium during the luteal-follicular transition. Endocrinology.

[44] McClellan MC, Rankin S, West NB, Brenner RM (1990). Estrogen receptors, progestin receptors and DNA synthesis in the macaque endometrium during the luteal-follicular transition. J Steroid Biochem Mol Biol.

[45] Brenner RM, Slayden OD (2004). Steroid receptors in blood vessels of the rhesus macaque endometrium: a review. Arch Histol Cytol.

[46] Brenner RM, Slayden OD, Nayak NR, Baird DT, Critchley HO (2003). A role for the androgen receptor in the endometrial antiproliferative effects of progesterone antagonists. Steroids.

[47] Slayden OD, Brenner RM (2004). Hormonal regulation and localization of estrogen, progestin and androgen receptors in the endometrium of nonhuman primates: effects of progesterone receptor antagonists. Arch Histol Cytol.

[48] Critchley HOD, Brenner RM, Henderson TA, Williams K, Nayak NR, Slayden OD (2001). Estrogen receptor beta, but not estrogen receptor alpha, is present in the vascular endothelium of the human and nonhuman primate endometrium. J Clin Endocrinol Metab.

[49] Markee JE (1940). Menstruation in intraocular endometrial transplants in the rhesus monkey. Contrib Embryol.

[50] Markee JE (1948). Morphological basis for menstrual bleeding. Bull N Y Acad Med.

[51] Nayak NR, Brenner RM (2002). Vascular proliferation and vascular endothelial growth factor expression in the rhesus macaque endometrium. J Clin Endocrinol Metab.

[52] Nayak NR, Kuo CJ, Desai TA, Wiegand SJ, Lasley BL, Giudice LC (2005). Expression, localization and hormonal control of angiopoietin-1 in the rhesus macaque endometrium: potential role in spiral artery growth. Mol Hum Reprod.

[53] Bausero P, Cavaillé F, Méduri G, Freitas S, Perrot-Applanat M (1998). Paracrine action of vascular endothelial growth factor in the human endometrium: production and target sites, and hormonal regulation. Angiogenesis.

[54] Fraser IS, McCarron G, Hutton B, Macey D (1987). Endometrial blood flow measured by xenon 133 clearance in women with normal menstrual cycles and dysfunctional uterine bleeding. Am J Obstet Gynecol.

[55] Enders AC, King BF (1991). Early stages of trophoblastic invasion of the maternal vascular system during implantation in the macaque and baboon. Am J Anat.

[56] Keator CS, Mah K, Slayden OD (2012). Alterations in progesterone receptor membrane component 2 (PGRMC2) in the endometrium of macaques afflicted with advanced endometriosis. Mol Hum Reprod.

[57] Markee JE, Meigs JV, Surgis SH (1950). The morphological and endocrine basis for menstrual bleeding. Progress in Gynecology.

[58] Rodgers WH, Matrisian LM, Giudice LC, Dsupin B, Cannon P, Svitek C (1994). Patterns of matrix metalloproteinase expression in cycling endometrium imply differential functions and regulation by steroid hormones. J Clin Investig.

[59] Salamonsen LA, Butt AR, Hammond FR, Garcia C, Zhang J (1997). Production of endometrial matrix metalloproteinases, but not their tissue inhibitors, is modulated by progesterone withdrawal in an *in vitro* model for menstruation. J Clin Endocrinol Metab.

[60] Zhang J, Salamonsen LA (2002). *In vivo* evidence for active matrix metalloproteinases in human endometrium supports their role in tissue breakdown at menstruation. J Clin Endocrinol Metab.

[61] Birkedal-Hansen H, Moore WG, Bodden MK, Windsor LJ, Birkedal-Hansen B, DeCarlo A (1993). Matrix metalloproteinases: a review. Crit Rev Oral Biol Med.

[62] Cox KE, Piva M, Sharpe-Timms KL (2001). Differential regulation of matrix metalloproteinase-3 gene expression in endometriotic lesions compared with endometrium. Biol Reprod.

[63] Nothnick WB (2000). Disruption of the tissue inhibitor of metalloproteinase-1 gene results in altered reproductive cyclicity and uterine morphology in reproductive-age female mice. Biol Reprod.

[64] Marbaix E, Donnez J, Courtoy PJ, Eeckhout Y (1992). Progesterone regulates the activity of collagenase and related gelatinases A and B in human endometrial explants. Proc Natl Acad Sci U S A.

[65] Bruner KL, Rodgers WH, Gold LI, Korc M, Hargrove JT, Matrisian LM (1995). Transforming growth factor β mediates the progesterone suppression of an epithelial metalloproteinase by adjacent stroma in the human endometrium. Proc Natl Acad Sci U S A.

[66] Cornet PB, Picquet C, Lemoine P, Osteen KG, Bruner-Tran KL, Tabibzadeh S (2002). Regulation and function of LEFTY-A/EBAF in the human endometrium. mRNA expression during the menstrual cycle, control by progesterone, and effect on matrix metalloprotineases. J Biol Chem.

[67] Tabibzadeh S (2002). Decoding implantation and menstruation: the tale of two opposing signals. Front Biosci.

[68] Kelly RW, King AE, Critchley HOD (2001). Cytokine control in human endometrium. Reproduction.

[69] Kelly RW, King AE, Critchley HO (2002). Inflammatory mediators and endometrial function–focus on the perivascular cell. J Reprod Immunol.

[70] Sharkey AM, Day K, Mcpherson A, Malik S, Licence D, Smith SK (2000). Vascular endothelial growth factor expression in human endometrium is regulated by hypoxia. J Clin Endocrinol Metab.

[71] Ferrara N, Davis-Smyth T (1997). The biology of vascular endothelial growth factor. Endocr Rev.

[72] Klagsbrun M, D’Amore PA (1996). Vascular endothelial growth factor and its receptors. Cytokine Growth Factor Rev.

[73] Ludwig H, Spornitz UM (1991). Microarchitecture of the human endometrium by scanning electron microscopy: menstrual desquamation and remodeling. Ann N Y Acad Sci.

[74] O’Toole EA (2001). Extracellular matrix and keratinocyte migration. Clin Exp Dermatol.

[75] Kim JP, Zang K, Kramer RH, Schall TJ, Woodley DT (1992). Integrin receptors and RGD sequences in human keratinocyte migration: unique anti-migratory function of alpha 3 beta 1 epiligrin receptor. J Investig Dermatol.

[76] Livant DL, Brabec RK, Kurachi K, Allen DL, Wu Y, Haaseth R (2000). The PHSRN sequence induces extracellular matrix invasion and accelerates wound healing in obese diabetic mice. J Clin Investig.

[77] Gehlsen KR, Argraves WS (1988). Inhibition of *in vitro* tumor cell invasion by Art-Gly-Asp-containing synthetic peptides. J Cell Biol.

[78] Cao W, Mah K, Carroll RS, Slayden OD, Brenner RM (2007). Progesterone withdrawal upregulates fibronectin and integrins during menstruation and repair in the rhesus macaque endometrium. Hum Reprod.

